# Outcomes and Challenges of Redo Coronary Artery Bypass Grafting: A Propensity-Matched Comparative Analysis

**DOI:** 10.21470/1678-9741-2025-0202

**Published:** 2026-09-08

**Authors:** Ahmet Arnaz, Vusal Mahmudov, Yusuf Kenan Yalcinbas

**Affiliations:** 1 Department of Cardiovascular Surgery, Acibadem University School of Medicine, Istanbul, Turkey.; 2 Department of Cardiovascular Surgery, Acibadem Bakirkoy Hospital, Istanbul, Turkey.

**Keywords:** Repeat Coronary Artery Bypass, Cardiac Surgery Outcomes, Revascularization Strategy, Long-Term Survival, Propensity Score Matching

## Abstract

**Introduction:**

This study aims to compare the perioperative and long-term outcomes of reoperative coronary artery bypass grafting (CABG) with primary CABG in a propensity score-matched patient cohort.

**Methods:**

We retrospectively analyzed 2,021 patients who underwent CABG between January 2003 and January 2024. Among them, 35 (1.7%) patients underwent redo CABG. Using 1:1 propensity score matching based on age, ejection fraction, diabetes mellitus, and hypertension, 36 patients who underwent primary CABG were selected for comparison. Patients with incomplete records or combined cardiac procedures were excluded. Perioperative variables, in-hospital mortality, and long-term survival were analyzed.

**Results:**

The redo CABG group had significantly longer intensive care unit stays (2.15 ± 2.37 vs. 1.39 ± 1.36 days, *P* = 0.006), hospital stays (12.11 ± 17.40 vs. 7.36 ± 2.21 days, *P* = 0.009), and postoperative bleeding (899.12 ± 445.17 vs. 576.39 ± 323.92 ml, *P* = 0.001). Hospital mortality occurred in two patients (5.7%) in the redo group and one patient (2.8%) in the primary group (*P* = 0.614). Kaplan-Meier survival analysis showed higher long-term survival in the redo CABG group (77.1%) compared to the primary CABG group (58.3%), though not statistically significant (*P* = 0.168).

**Conclusion:**

Despite the increased perioperative complexity, redo CABG is associated with low in-hospital mortality and promising long-term outcomes when appropriate patient selection and meticulous surgical technique are employed. These findings are especially relevant in guiding revascularization strategies for patients with prior CABG, where redo surgery is often underutilized despite its clinical viability.

## INTRODUCTION

Coronary artery disease (CAD) remains a leading cause of morbidity and mortality worldwide, despite advancements in medical, interventional, and surgical therapies. Coronary artery bypass grafting (CABG) is a well-established treatment for severe forms of CAD. As the population ages and the longevity of patients with previous CABG increases, a growing number of patients are presenting with the need for coronary artery reoperations despite the improvement in medical treatment and percutaneous coronary interventions (PCI). Reoperative CABG presents unique challenges and risks compared to first-time CABG procedures, including increased operative mortality and morbidity^[1,2]^.

The decision for reoperation involves careful consideration of patient-specific factors and the evolving understanding of long-term outcomes post-revascularization. Technological advances and improvements in surgical techniques have contributed to enhanced outcomes and expanded indications for reoperation, potentially offering improved quality of life and survival benefits for selected patients^[3]^.

Moreover, the comparison between reoperative CABG and first-time CABG procedures is critical for understanding the differential impacts on patient outcomes and healthcare resources. This comparison also underscores the importance of initial graft selection and operative strategy during the first-time CABG, which may influence the ease and safety of any subsequent reoperation^[4]^.

This study aims to provide a comprehensive comparison of redo *vs.* primary CABG, evaluating perioperative parameters and long-term outcomes through a propensity-matched analysis, thereby offering insights into real-world management strategies and guiding clinical decision-making.

## METHODS

### Study Population

A total of 2,021 patients who underwent CABG between January 2003 and January 2024 were retrospectively reviewed. Among these, 35 patients (1.7%) underwent redo CABG and were included in the study group. To ensure comparability, 36 patients who underwent primary CABG were selected through 1:1 propensity score matching based on age, ejection fraction (EF), diabetes mellitus, and hypertension. Patients with incomplete data or combined cardiac procedures were excluded from the analysis.

This study was approved by the Acibadem University Medical Research Ethics Committee (ATADEK) under protocol number ATADEK-2024/17, with decision number 2024-17/659, dated October 31, 2024. The study was conducted in accordance with the Declaration of Helsinki.

### Matching Criteria

Propensity score matching was performed using nearest-neighbor matching without replacement, applying a caliper width of 0.2. Sex was not included as a matching variable, as previous literature indicates a male predominance in redo CABG cases and excluding this variable maintains population representativeness.

### Data collection

Patient demographic data, clinical comorbidities (diabetes, hypertension, hyperlipidemia, renal function, EF), and lifestyle factors (*e.g.*, smoking status) were extracted from institutional records. Procedural variables included the number of distal anastomoses, graft types used (left internal mammary artery [LIMA], right internal mammary artery [RIMA], radial artery [RA], saphenous vein grafts [SVG]), cardiopulmonary bypass (CPB) and cross-clamping times, and intraoperative and postoperative outcomes including intensive care unit (ICU)/hospital stay duration, intubation time, blood product usage, infection rate, and bleeding volume.

### Surgical Technique

Redo CABG was performed via median sternotomy. Careful dissection minimized injury to prior grafts. Standard CPB and myocardial protection via cold blood cardioplegia followed by warm reperfusion were utilized. Arterial conduits were preferred when feasible.

### Statistical Analysis

All statistical analyses were performed using PASS 2020 Statistical Software (Utah, United States of America). Descriptive statistics included mean, standard deviation, median, frequency, and percentage. The Shapiro-Wilk test and box plot graphs were used to assess the normality of distribution. For non-normally distributed variables, comparisons between groups were made using the Mann-Whitney U test. Categorical variables were compared using the Pearson’s Chi-square test, Fisher’s exact test, and Fisher-Freeman-Halton test. A significance level of *P* < 0.05 was used to determine statistical significance.

## RESULTS

The demographic and clinical characteristics of the study population are summarized in Table 1. The cohort consisted of 71 patients, with a majority being male (n = 50, 70.4%) and the remaining being female (n = 21, 29.6%), between January 2003 and January 2020. The mean age of the patients was 61.94 ± 9.04 years, with a median age of 61 years, ranging from 43 to 80 years.

**Table 1.** Distribution of descriptive features.

		Total (n = 71)	Redo CABG (n = 35)	First-time CABG (n = 36)	*P*-value
Sex	Male	50 (70.4)	31 (88.6)	19 (52.8)	^a*^0.001
	Female	21 (29.6)	4 (11.4)	17 (47.2)	
Age	Mean ± SD	61.94 ± 9.04	62.37 ± 9.16	61.53 ± 9.05	^t^0.697
	Median (min - max)	61 (43 - 80)	62 (46 - 80)	59.5 (43 - 79)	
Diabetes mellitus		34 (47.9)	13 (37.1)	21 (858.3)	^a^0.074
Hyperlipidemia		14 (19.7)	9 (25.7)	5 (13.9)	^a^0.211
Hypertension		43 (60.6)	1 (54.3)	24 (66.7)	^a^0.286
Smoking		19 (26.8)	11 (31.4)	8 (22.2)	^a^0.381
Creatinine	Mean ± SD	0.92 ± 0.30	0.97 ± 0.28	0.87 ± 0.32	^d^0.080
	Median (min - max)	0.86 (0.5 - 2.3)	0.9 (0.5 – 1.7)	0.85 (0.5 – 2.3)	
Ejection fraction	Normal	47 (66.2)	22 (62.9)	25 (69,4)	^c^0.685
	Moderate	19 (26.8)	11(31.4)	8 (22,2)	
	Low	5 (7.0)	2 (5.7)	3 (8,3)	
Follow-up duration	Mean ± SD	171.94 ± 64.39	170.58 ± 71.65	173.26 ± 57.46	^t^0.862
	Median (min - max)	181.3 (0.03 - 258.7)	173.98 (0.03 – 256.7)	182,1 (0,07 – 258.7)	
Hospital mortality		3 (4.2)	2 (5.7)	1 (2.8)	^aa^0.614

a Pearson’s Chi-square test;

aa Fisher’s exact test;

c Fisher-Freeman-Halton test;

d Mann-Whitney U test;

t Student’s *t*-test;

* *P* < 0.01

CABG=coronary artery bypass grafting; SD=standard deviation

The patients were categorized into two groups based on their surgical history: those undergoing a repeat CABG constituted 49.3% (n = 35) of the cohort, while those undergoing their first-time CABG comprised 50.7% (n = 36) of the study population. The time interval between the initial CABG surgery and the redo CABG surgery was evaluated for the 35 patients in the redo group. The mean time between surgeries was 12.2 ± 6.1 years, with a range from one to 27 years. The median time interval was 12 years.

The comparison of the baseline characteristics between patients who underwent redo CABG and those who underwent first-time CABG are presented in Table 1.

The sex distribution was significantly different between the two groups, with the majority of patients in the redo CABG group being male (88.6%), compared to 52.8% in the first-time CABG group (*P* = 0.001). The mean age was similar between the two groups, with no statistically significant difference (*P* = 0.697), and the median ages also closely aligned.

In terms of comorbidities, the prevalence of diabetes mellitus was higher in the first-time CABG group (58.3%) compared to the redo CABG group (37.1%), although this difference did not reach statistical significance (*P* = 0.074). Similarly, no significant differences were observed in the prevalence of hyperlipidemia (*P* = 0.211) or hypertension (*P* = 0.286) between the two groups. Smoking status was also comparable, with 31.4% of patients in the redo CABG group and 22.2% in the first-time CABG group being smokers (*P* = 0.381).

Renal function, as measured by creatinine levels, showed a trend towards higher creatinine levels in the redo CABG group, but this difference was not statistically significant (*P* = 0.080). The median creatinine levels were similar between the groups. EF, which is a measure of cardiac function, was largely normal across both groups, with no significant differences in the distribution of normal, moderate, and low EF (*P* = 0.685).

The follow-up duration was also similar between the groups, with no statistically significant difference observed in the mean or median follow-up times (*P* = 0.862). Hospital mortality occurred in three patients overall (4.2%), including two patients in the redo CABG group (5.7%) and one patient in the primary CABG group (2.8%), with no statistically significant difference between the groups (*P* = 0.614).

The comparison of patients undergoing redo CABG and first-time CABG based on the number of distal anastomoses and the types of grafts used during the surgery are shown in Table 2.

**Table 2.** Number of distal anastomosis and graft types according to groups.

		Redo CABG (n = 35)	First-time CABG (n = 36)	*P*-value
CABG (distal anastomoses)	1	5 (14.3)	1 (2.8)	^c^0.128
	2	14 (40)	10 (27.8)	
	3	11 (31.4)	13 (36.1)	
	4	3 (8.6)	8 (22.2)	
	5	1 (2.9)	4 (11.1)	
	6	1 (2.9)	0 (0)	
Grafts used				
RA	Absent	27 (77.1)	33 (91.7)	^a^0.091
	Present	8 (22.9)	3 (8.3)	
LIMA	Absent	31 (88.6)	3 (8.3)	^a*^0.001
	Present	4 (11.4)	33 (91.7)	
RIMA	Absent	33 (94.3)	36 (100)	^aa^0.239
	Present	2 (5.7)	0 (0)	
SVG	Absent	6 (17.1)	2 (5.6)	^aa^0.151
	Present	29 (82.9)	34 (94.4)	

a Pearson’s Chi-square test;

aa Fisher’s exact test;

c Fisher-Freeman-Halton test;

* *P* < 0.01

CABG=coronary artery bypass grafting; LIMA=left internal mammary artery; RA=radial artery; RIMA=right internal mammary artery; SVG=saphenous vein grafts

When comparing the number of distal anastomoses, it is observed that most patients had between two and three anastomoses in both groups, with no statistically significant difference (*P* = 0.128). In the redo CABG group, 40% of patients had two anastomoses, compared to 27.8% in the first-time CABG group. Similarly, 36.1% of patients in the first-time CABG group had three anastomoses, compared to 31.4% in the first-time CABG group.

Regarding the use of different graft types, the RA was used more frequently in the redo CABG group (22.9%) compared to the first-time CABG group (8.3%), though this difference was not statistically significant (*P* = 0.091). The use of LIMA was significantly different between the groups (*P* = 0.001), with 91.7% of patients in the first-time CABG group receiving a LIMA graft, compared to only 11.4% in the redo CABG group. This highlights the more frequent use of LIMA grafts in first-time CABG procedures. The use of RIMA was rare in both groups, with only 5.7% of patients in the redo CABG group receiving a RIMA graft. None of the patients in the first-time CABG group underwent RIMA grafting, and this difference was not statistically significant (*P* = 0.239). Lastly, saphenous vein grafts SVG were used more frequently in the first-time CABG group (94.4%) compared to the redo CABG group (82.9%). However, this difference was not statistically significant (*P* = 0.151). The lower usage of LIMA in the redo CABG group can be attributed to the fact that LIMA was commonly utilized in the first operation, making it unavailable for use in subsequent surgeries. In contrast, RA and RIMA are more frequently used in redo CABG procedures, either as an alternative arterial graft or due to limited graft options in the second operation.

The comparison of outcome variables between patients undergoing redo CABG and those undergoing first-time CABG showed several significant differences (Table 3).

**Table 3.** Comparison of outcome variables by CABG applications.

		Redo CABG	First-time CABG	*P*-value
		(n = 35)	(n = 36)	
ICU stay (days)	Mean ± SD	2.15 ± 2.37	1.39 ± 1.36	^d**^0.006
	Median (min - max)	1.50 (1 - 14)	1 (1 - 9)	
Hospital stay (days)	Mean ± SD	12.11 ± 17.40	7.39 ± 2.21	^d*^*0.009
	Median (min - max)	8 (3 - 109)	7 (2 - 15)	
CPB (minutes)	Mean ± SD	119.27 ± 60.78	83.86 ± 28.87	^**^0.002
	Median (min - max)	109 (36 - 369)	8 (35 - 150)	
Cross-clamping time (minutes)	Mean ± SD	66.53 ± 30.86	50.71 ± 23.59	^*^0.016
	Median (min - max)	65 (20 - 154)	46 (17 - 117)	
Total blood product usage	Mean ± SD	2.66 ± 2.73	1.00 ± 1.60	^**^0.001
	Median (min - max)	2 (0 - 12)	0.5 (0 - 8)	
Intubation duration (hours)	Mean ± SD	11.24 ± 5.95	9.53 ± 5.56	^d^0.199
	Median (min - max)	10 (4 - 35)	9 (4 - 40)	
Bleeding volume (ml)	Mean ± SD	899.12 ± 445.17	576.39 ± 323.92	^d**^0.001
	Median (min - max)	775 (350 - 2510)	475 (200 - 1850)	
Infection	None	32 (94.1)	36 (100)	^a^0.232
	Yes	2 (5.9)	0	
Follow-up (months)	Mean ± SD	170.59 ± 71.66	173.26 ± 57.46	^d^0.814
	Median (min - max)	173.98 (0.03 – 256.7)	182.09 (0.07 – 258.67)	

a Pearson’s Chi-square test;

d Mann-Whitney U test;

* *P* < 0.05;

** *P* < 0.01

CABG=coronary artery bypass grafting; CPB=cardiopulmonary bypass; ICU=intensive care unit; SD=standard deviation

Patients who underwent redo CABG had significantly longer stays in the ICU compared to those who underwent first-time CABG, with a mean ICU stay of 2.15 ± 2.37 days in the redo CABG group *vs.* 1.39 ± 1.36 days in the first-time CABG group (*P* = 0.006) (Figure 1). Similarly, the total hospital stay was also significantly longer in the redo CABG group, with a mean of 12.11 ± 17.40 days compared to 7.36 ± 2.21 days in the first-time CABG group (*P* = 0.009) (Figure 2).

**Fig. 1:**
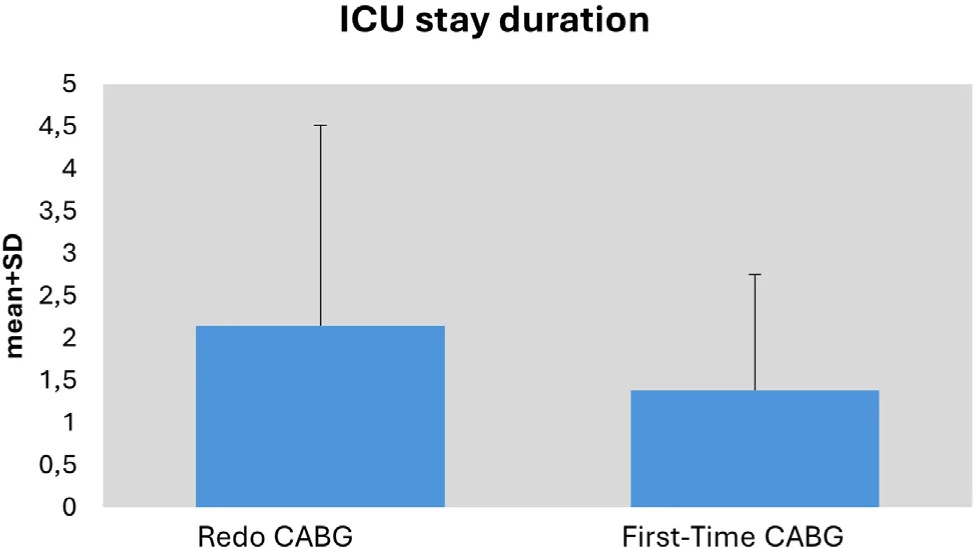
Distribution of intensive care unit (ICU) periods by groups. CABG=coronary artery bypass grafting; SD=standard deviation.

**Fig. 2:**
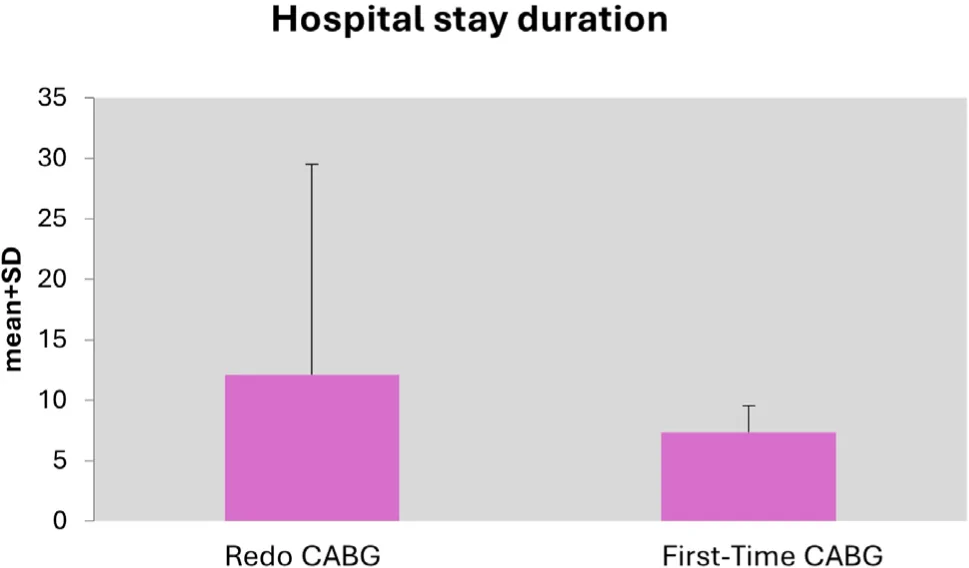
Distribution of hospital stay by groups. CABG=coronary artery bypass grafting; SD=standard deviation.

When examining CPB time, the redo CABG group had a significantly longer mean CPB time of 119.27 ± 60.78 minutes compared to 83.86 ± 28.87 minutes in the first-time CABG group (*P* = 0.002). Additionally, the cross-clamping time was significantly longer in the redo CABG group, with a mean time of 66.53 ± 30.86 minutes *vs.* 50.71 ± 23.59 minutes in the first-time CABG group (*P* = 0.016).

The total blood product usage was notably higher in the redo CABG group, with a mean of 2.66 ± 2.73 units compared to 1.00 ± 1.60 units in the first-time CABG group (*P* = 0.001).

Although the intubation time was longer in the redo CABG group (11.24 ± 5.95 hours) compared to the first-time CABG group (9.53 ± 5.56 hours), this difference was not statistically significant (*P* = 0.199). The bleeding volume during the postoperative period was significantly higher in the redo CABG group, with a mean volume of 899.12 ± 445.17 ml compared to 576.39 ± 323.92 ml in the first-time CABG group (*P* = 0.001). (Figure 3) The rate of infections was low in both groups, with 5.9% of patients in the redo CABG group experiencing infections, compared to none in the first-time CABG group. However, this difference was not statistically significant (*P* = 0.232). Lastly, the follow-up duration was comparable between the two groups, with no statistically significant difference observed (*P* = 0.814).

**Fig. 3:**
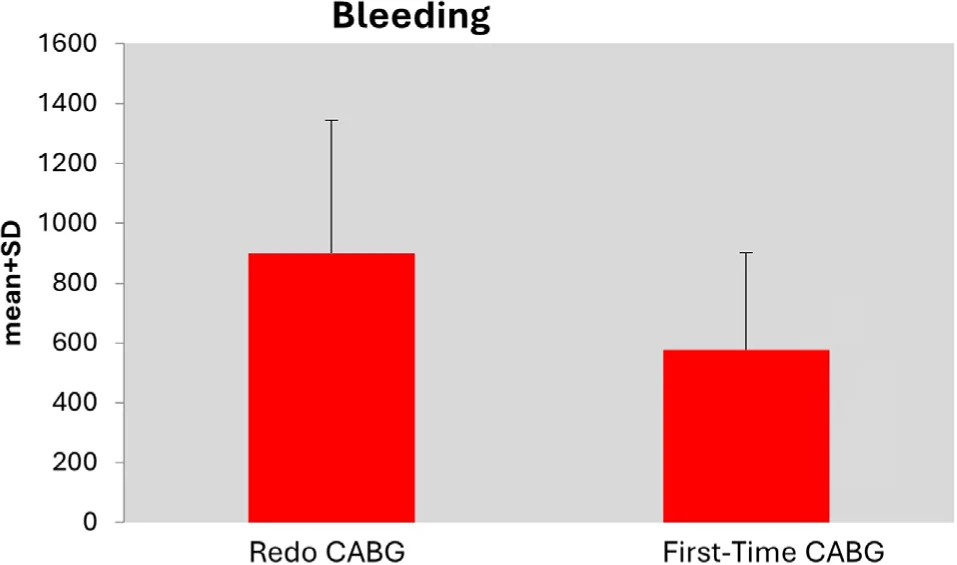
Distribution of bleeding amounts by groups. CABG=coronary artery bypass grafting; SD=standard deviation.

These results highlight the increased complexity and risks associated with redo CABG procedures compared to first-time CABG surgeries.

### Overall Survival Analysis (By Groups)

The survival analysis presented in Table 4 shows a clear difference in the survival rates between the two groups: the redo CABG group had a survival rate of 77.1% compared to 58.3% in the first-time CABG group. However, despite the apparent difference in survival percentages, the log-rank test suggests that this difference is not statistically significant (*P* = 0.168).

**Table 4.** Survival analysis by groups.

	N	Late mortality (n)	Surviving patients (n)	Survival rate (%)	Mean survival time (months)	95% Confidence interval	*P*-value
						Lower-upper	
Redo CABG	35	8	27	77.1%	217.136 ± 13.017	191.623 – 242.649	0.168
First-time CABG	36	15	21	58.3%	207.170 ± 10.154	187.268 – 227.072	

Kaplan-Meier analysis

CABG=coronary artery bypass grafting

The Kaplan-Meier analysis further reveals that the last recorded deaths occurred later in the redo CABG group (19.8 years) compared to the first-time CABG group (18.1 years), which might indicate a slightly longer survival for redo CABG patients. Nevertheless, the overall results imply that the observed differences could be due to chance rather than a definitive difference between the two surgical approaches.

This analysis highlights the importance of continued follow-up and larger sample sizes to better understand the long-term outcomes of redo CABG compared to first-time CABG, as the current results do not provide conclusive evidence of a survival advantage for either group.

In both groups, survival rates initially remain high and close to 1.0 in the early follow-up period. As time progresses, a steeper decline in the survival rate is observed in the first-time CABG group compared to the redo CABG group. Around the 150-month mark, the first-time CABG group shows a more pronounced decrease in cumulative survival.

By the end of the observation period, the cumulative survival probability in the redo CABG group remains higher, with several patients still alive past the 200-month mark. In contrast, survival in the first-time CABG group declines more significantly, indicating a lower long-term survival rate.

The censored observations, represented by "+" symbols on the curves, indicate the points at which patients were either lost to follow-up or survived beyond the last observed event (Figure 4).

**Fig. 4:**
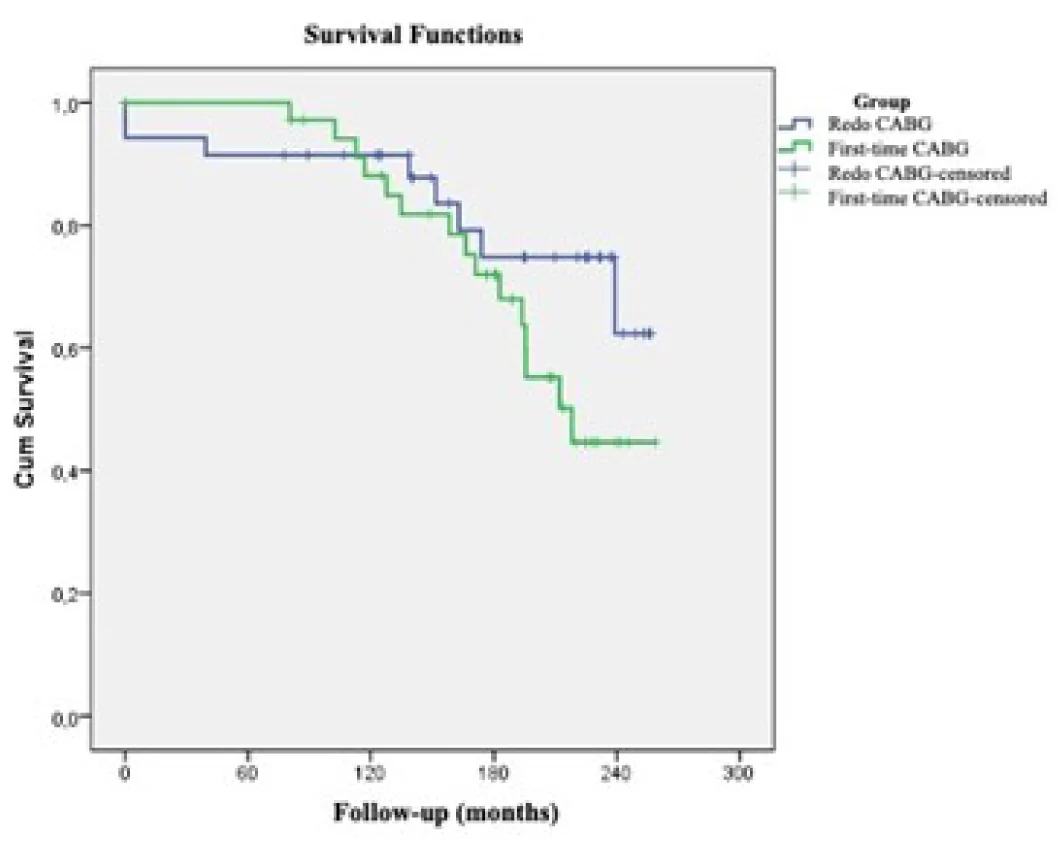
Survival curve for redo coronary artery bypass grafting (CABG) and first-time CABG groups.

Despite the visible difference between the survival curves, the log-rank test did not reveal a statistically significant difference in survival between the two groups (*P* = 0.168). This suggests that, while the survival trend favors the redo CABG group, the difference may not be robust enough to conclude a definitive survival advantage in this sample.

## DISCUSSION

Redo CABG is a technically challenging procedure that carries higher risks compared to primary CABG. Patients undergoing redo CABG are generally older and present with more comorbidities, including advanced CAD, reduced ventricular function, and a history of myocardial infarction or previous PCI. These factors contribute to the increased complexity and risk of complications during redo CABG^[5,6]^.

The incidence of redo CABG has decreased over time, largely due to improvements in primary CABG techniques, such as the routine use of the left internal thoracic artery for grafting the left anterior descending artery, and advancements in PCI and medical therapies^[5]^. Despite this, redo CABG remains necessary for a subset of patients who experience graft failure or progression of CAD. Our study aimed to evaluate the outcomes of redo CABG in comparison to primary CABG, with a focus on perioperative and long-term results.

Our analysis demonstrated that patients undergoing redo CABG tend to have a higher risk profile, including increased comorbidities such as diabetes, hypertension, and dyslipidaemia. This aligns with the findings of Elbadawi et al.^[6]^, who highlighted that redo CABG patients often present with more advanced comorbid conditions compared to those undergoing primary CABG. Additionally, our study noted a longer ICU stay and hospital stay for redo CABG patients, reflecting the increased complexity and postoperative care required for these patients. The increased duration of ICU and hospital stays in redo CABG patients compared to first-time CABG patients is consistent with previous findings. For instance, studies by Gaudino et al.^[7]^ and Elbadawi et al.^[6]^ also reported longer hospital stays and higher resource utilization for redo CABG patients^[6-9]^. Our findings further support these observations, with redo CABG patients showing significantly longer ICU stays (mean 2.15 ± 2.37 days *vs.* 1.39 ± 1.36 days, *P* = 0.006) and hospital stays (mean 12.11 ± 17.40 days *vs.* 7.36 ± 9.21 days, *P* = 0.009).

The relatively small number of redo CABG patients in our 21-year cohort may seem limited; however, this reflects the declining global trend in redo CABG surgeries due to improvements in primary surgical techniques, graft durability, and widespread use of PCI. Contemporary data suggest that redo CABG accounts for < 5% of all coronary revascularization procedures^[5]^.

The sex distribution imbalance observed between the groups, with a higher male ratio in the redo CABG group, is consistent with the literature, as male patients are more likely to undergo reintervention due to earlier onset and more aggressive progression of CAD. Therefore, sex was not included in the propensity score matching process to preserve the clinical representativeness of the redo CABG population^[6]^.

One of the critical issues with redo CABG is the increased risk of postoperative complications. Our study observed a significantly higher amount of bleeding in redo CABG patients (mean 899.12 ± 445.17 ml *vs.* 576.39 ± 323.92 ml, *P* = 0.001). This finding is consistent with the literature, which indicates that the technical challenges of redo CABG, such as the presence of adhesions and the need for more extensive dissection, contribute to higher intraoperative and postoperative bleeding risks^[10,11]^.

Despite the increased risks, the outcomes in terms of in-hospital mortality have improved over the years. Historical data from the 1990s reported much higher operative mortality rates for redo CABG, often exceeding 7% to 10%. However, more recent studies, including our own, indicate a significant reduction in mortality rates, reflecting advancements in surgical techniques and perioperative care. Our study reported an in-hospital mortality rate of 3.1% for redo CABG patients, which is in line with the findings of Elbadawi et al.^[6]^ and other contemporary analyses^[8]^.

Our study also explored the implications of graft selection in redo CABG. The use of arterial grafts, such as LIMA, was significantly higher in first-time CABG patients compared to redo CABG patients. This difference is critical as arterial grafts are associated with better long-term patency rates and outcomes. However, the technical difficulties in using arterial grafts in redo surgeries often necessitate alternative strategies.

The long-term outcomes for patients undergoing redo CABG are generally poorer compared to those undergoing primary CABG. Studies have reported worse five-year clinical outcomes for redo patients, with lower freedom from death of any cause, cardiac death, myocardial infarction, and other cardiac event. This can be attributed to the fact that patients requiring redo CABG often have more advanced CAD and other comorbid conditions that complicate their postoperative recovery and long-term prognosis^[12-14]^. However, in contrast to these findings, our study demonstrated higher long-term survival rates for patients in the redo CABG group compared to the first-time CABG group. Although this difference did not reach statistical significance (log-rank *P* = 0.168), the survival curve tended to favor the redo CABG group, particularly beyond the 150-month follow-up mark. The redo CABG group showed a 77.1% survival rate *vs.* 58.3% in the first-time CABG group. Although the observed survival advantage may have been influenced by sample size and baseline heterogeneity, the trend toward improved long-term outcomes in redo CABG patients challenges the conventional perception that reoperative CABG is inherently associated with worse prognosis. This finding may warrant further investigation in larger, multicenter studies and should be considered during surgical decision-making processes.

Recent advances in surgical techniques, including the use of off-pump CABG and minimally invasive procedures, have shown promise in reducing some of the risks associated with redo CABG. Off-pump techniques have been associated with reduced incidences of incomplete revascularization and myocardial injury during the surgery. These advancements are crucial in improving the outcomes for patients undergoing redo CABG^[11,15,16]^.

In patients who require revascularization after previous coronary CABG, the decision between undergoing repeat CABG or opting for PCI is complex and necessitates a careful evaluation of patient-specific factors. Repeat CABG is associated with the advantage of complete revascularization, which is crucial for long-term outcomes; however, it also carries significantly higher operative risks, particularly in older patients or those with comorbidities such as diabetes, hypertension, and renal disease^[17]^. On the other hand, PCI is less invasive and offers lower short-term mortality, making it an attractive alternative in certain cases. However, PCI may be limited by its potential for incomplete revascularization, especially when treating graft lesions, which can result in higher rates of restenosis^[18]^. The decision-making process should, therefore, be individualized, considering the patient's overall risk profile, the extent of CAD, and the technical feasibility of achieving complete revascularization with either strategy. Multidisciplinary collaboration and the use of advanced imaging and interventional techniques can aid in optimizing the treatment approach for this challenging patient population.

In clinical practice, patients with prior CABG are often referred to PCI due to the perceived risks of redo surgery. This tendency is particularly pronounced among cardiologists, who may be reluctant to refer patients to redo CABG due to concerns over operative complexity, higher perioperative risk, and perceived surgical morbidity. However, our findings suggest that, when patients are appropriately selected, redo CABG can be performed with acceptable perioperative risk and favorable long-term survival — comparable to primary CABG. This highlights the need for a multidisciplinary heart team approach in decision-making, particularly in cases where complete revascularization is desired. Our study supports the view that redo CABG remains a viable and underutilized option in selected patients, and this perspective should be reflected in institutional and guideline-based revascularization strategies.

### Limitations

This study has some limitations due to its retrospective design and single-center nature. Moreover, the relatively small sample size may limit the generalizability of our findings. While sex was not included in the matching variables to preserve clinical representativeness, the observed imbalance may introduce a potential confounding effect and should be acknowledged as a limitation. Although redo CABG is often considered technically more challenging due to adhesions and the risk of graft injury, in our cohort, no major intraoperative bleeding or injury to previous grafts was encountered during re-sternotomy and dissection. This may reflect both careful preoperative planning and surgical experience.

## CONCLUSION

Redo CABG is associated with greater perioperative resource utilization and surgical complexity, as reflected by longer cardiopulmonary bypass and cross-clamp times, increased postoperative bleeding and blood product requirements, and prolonged ICU and hospital stays. Nevertheless, in this propensity-matched cohort, in-hospital mortality did not differ significantly from that observed after primary CABG. Long-term survival was numerically higher in the redo CABG group, although this difference was not statistically significant. These findings suggest that redo CABG remains a feasible revascularization option in appropriately selected patients, provided that its increased perioperative burden is recognized. Larger multicenter studies are required to further define its long-term comparative outcomes.

## Data Availability

The authors declare that anonymized data will be available upon request to the authors and with the approval of the Acibadem University Ethics Committee.
